# Randomised, double-blind, placebo-controlled crossover study to investigate different dosing regimens of olodaterol delivered via Respimat® in patients with moderate to severe persistent asthma

**DOI:** 10.1186/s12931-015-0243-1

**Published:** 2015-07-16

**Authors:** Kai-Michael Beeh, Craig LaForce, Martina Gahlemann, Arne Wenz, Robert Toorawa, Matjaž Fležar

**Affiliations:** Insaf GmbH Institut für Atemwegsforschung, Biebricher Allee 34, D-65187 Wiesbaden, Germany; North Carolina Clinical Research, Raleigh, NC USA; Boehringer Ingelheim Pharma GmbH & Co. KG, Biberach an der Riss, Germany; Boehringer Ingelheim (Schweiz) GmbH, Basel, Switzerland; Boehringer Ingelheim Ltd, Bracknell, Berkshire UK; University Clinic of Respiratory and Allergic Diseases, Golnik, Slovenia

**Keywords:** Olodaterol, Long-acting beta-2-agonist, Asthma

## Abstract

**Background:**

A Phase II, multicentre, randomised, double-blind, placebo-controlled, crossover trial comparing the 24-h forced expiratory volume in 1 s (FEV_1_) time profile after 3 weeks’ treatment with once-daily (QD) or twice-daily (BID) olodaterol (at the same total daily dose) versus placebo delivered via Respimat® in patients with moderate to severe asthma.

**Methods:**

Patients were randomised to different sequences of olodaterol with 2-week washout, either as a total daily dose of 5 μg (5 μg QD [AM] or 2.5 μg BID) or placebo, or 10 μg (10 μg QD [AM] or 5 μg BID) or placebo. Primary end point was FEV_1_ area under the curve from 0 to 24 h (AUC_0–24_) response (defined as change from study baseline FEV_1_) after 3 weeks. Key secondary end points were FEV_1_ AUC_0–12_ and AUC_12–24_ responses.

**Results:**

Two hundred and six patients received treatment. All olodaterol treatments demonstrated statistically significant improvements in FEV_1_ AUC_0–24_ response at 3 weeks versus placebo (*p* < 0.0001); adjusted mean treatment difference versus placebo was 0.191 L for olodaterol 2.5 μg BID (95 % confidence interval [CI] 0.152, 0.229), 0.150 L for 5 μg QD (95 % CI 0.111, 0.189), 0.228 L for 5 μg BID (95 % CI 0.190, 0.266) and 0.209 L for 10 μg QD (95 % CI 0.170, 0.247). These results were supported by the key secondary end points. Olodaterol 5 μg QD provided numerically lower mean values for 24-h bronchodilation than olodaterol 2.5 μg BID (*p* = 0.0465), with no statistically significant difference between treatment with olodaterol 10 μg QD and 5 μg BID. No relevant differences in morning and evening peak expiratory flow or Asthma Control Questionnaire scores at 3 weeks were observed between different doses and regimens. Adverse events were generally mild to moderate and comparable between groups.

**Conclusions:**

All doses and dose frequencies provided adequate 24-h bronchodilation superior to placebo. Based on the results of this study, it would be reasonable to include both posologies of 5 μg olodaterol daily (5 μg QD or 2.5 μg BID, both delivered in two puffs per dose from the Respimat® inhaler) in subsequent studies. Further studies are necessary to confirm the optimum dosing regimen in asthma. No safety concerns were identified.

**Trial registration:**

ClinicalTrials.gov NCT01311661

**Electronic supplementary material:**

The online version of this article (doi:10.1186/s12931-015-0243-1) contains supplementary material, which is available to authorized users.

## Background

Long-acting β_2_-agonists (LABAs) are used in combination with inhaled corticosteroids (ICS) as controller medication for asthma, and have been shown to improve lung function and symptom scores, and reduce the risk of severe exacerbations [1]. Bronchodilators are also well established as treatment for chronic obstructive pulmonary disease (COPD); LABAs have demonstrated significant improvements in lung function, health-related quality of life and exacerbations in this setting [2, 3].

First-generation LABAs, such as formoterol and salmeterol [4, 5], have a 12-h duration of action that requires a twice-daily (BID) dosing schedule. More recently, research efforts have focused on the development of LABAs with a longer duration of action, which may allow for more convenient once-daily (QD) dosing, potentially improving adherence [6, 7]. Olodaterol is a novel LABA, characterised by high β_2_ selectivity and a near full-agonist profile at β_2_ adrenoceptors, with a duration of action over 24 h demonstrated by preclinical studies [8]. Effective 24-h bronchodilation with olodaterol has subsequently been confirmed by single-dose studies in both asthma and COPD [9, 10], along with Phase II studies in COPD investigating four doses of QD olodaterol [11] and a comparison of QD and BID dosing [12] and a large Phase III programme in COPD [13–16]. These data also demonstrated an acceptable tolerability profile and no safety concerns were identified.

Dosing and posology have been the focus of recent discussion in development of new bronchodilators. Firstly, the goal of defining the lowest effective dose to minimise safety risk is of particular importance in view of specific safety considerations for LABA use in asthma [17]. Secondly, while demonstration of 24-h duration of action affords the opportunity to consider QD dosing, assessment of lung function trough measurements alone may not be sufficient to demonstrate that QD dosing is the most appropriate regimen. Specific clinical comparison of different doses and posologies (e.g. QD versus BID) over a 24-h period is the most appropriate method for determining the optimum dose and dosing schedule. The inhaled anticholinergic aclidinium bromide serves as a recent example of a drug developed as a QD treatment [18] but then reassessed at later stages of clinical development and finally approved as a BID drug at a different dose [19].

This study has been designed as a posology study for olodaterol in asthma in combination with maintenance therapy with ICS and forms part of a wider series of studies exploring the optimum dose and regimen of olodaterol in both asthma and COPD. One study investigated single-dose olodaterol [9] while two Phase II dose-finding studies were conducted using different designs in moderate asthma. One 4-week, parallel-group study (NCT00467740) demonstrated significant benefits with olodaterol versus placebo, and dose ordering with regards to peak expiratory flow (PEF) but not in forced expiratory volume in 1 s (FEV_1_), the primary end point of the study [20]. A subsequent incomplete-block crossover study with 4-week treatment periods (NCT01013753) reported significant benefits with olodaterol compared to placebo, and dose ordering observed with FEV_1_ and PEF [21].

This paper describes the results of our study that was designed to investigate dosing frequencies by comparing the 24-h FEV_1_ profile of the same olodaterol daily dose administered in either a QD or BID schedule, i.e. 5 μg QD in the morning versus 2.5 μg BID and 10 μg QD in the morning versus 5 μg BID in patients with moderate to severe persistent asthma on maintenance ICS. It is similar in design to a study carried out in patients with COPD (NCT00846768) [12] but with an additional placebo comparison. Total daily doses of 5 and 10 μg were chosen for evaluation as, based on an integrated view of earlier data for QD olodaterol, these doses are approaching the plateau on the dose-response curve [11, 21].

## Methods

### Patients

Patients were randomised if they met the following main inclusion criteria: aged ≥18 and ≤70 years; a diagnosis of moderate to severe asthma according to the Global Initiative for Asthma [22]; pre-bronchodilator FEV_1_ ≥60 % and <90 % of predicted FEV_1_; an increase in FEV_1_ of ≥12 % and ≥200 mL 15 min after administration of 400 μg salbutamol at screening visit; non-smokers or ex-smokers with a history of <10 pack-years (and smoking cessation ≥1 year prior to enrolment). Patients must have been taking ICS for ≥12 weeks prior to screening and a stable dose for >6 weeks (either medium- to high-dose ICS, or low- to high-dose ICS in fixed-dose combination with a LABA). Patients previously receiving fixed-dose combinations of LABA and ICS were required to demonstrate stability while continuing to receive the equivalent ICS monotherapy for ≥48 h prior to screening visit. Key exclusion criteria included: patients currently diagnosed with a significant disease other than asthma (determined by the investigator as any condition that may put the patient at risk if they entered the study, may influence the results of the study or may cause concern regarding the patient’s ability to participate in the study); patients who had been hospitalised for an asthma exacerbation within 3 months or admitted to an intensive care unit due to asthma within the past 3 years; and patients with a history of myocardial infarction, cor pulmonale or cystic fibrosis. The study was performed in accordance with the Declaration of Helsinki, International Conference on Harmonisation Good Clinical Practice Guidelines and local regulations. Prior to study initiation, the protocol was approved by the ethics research board of the respective institutions and signed consent was obtained from all patients.

The Independent Ethics Committee was Ethikkommittee der Landesärztekammer Hessen, Frankfurt am Main, Germany and the competent authority (Bundesinstitut für Arzneimittel und Medizinprodukte [BfArM], Bonn, Germany) approved the study on 23 Feb 2011.

### Study design

**Fig. 1:**
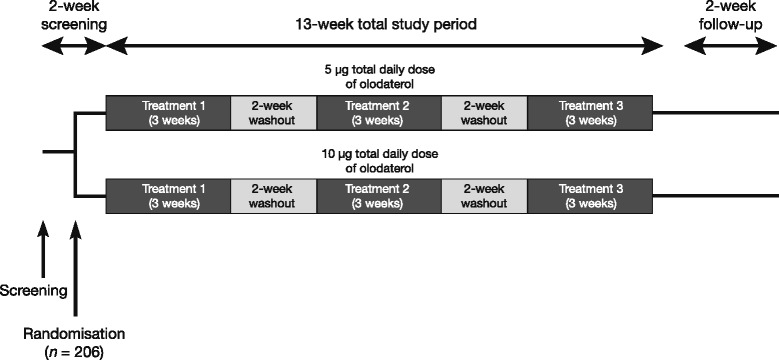
Study design. All patients received three dose regimens; each was separated by a 2-week washout period. Patients in the 5 μg total daily dose group received one of six possible sequences of olodaterol 2.5 μg BID, 5 μg QD and placebo. Patients in the 10 μg total daily dose group received one of six possible sequences of olodaterol 5 μg BID, 10 μg QD and placebo. Patients continued taking ICS throughout the study, with posology determined by former use. If administration of ICS and study treatment coincided, patients were to take study treatment followed by ICS

This was a Phase II, multicentre, randomised, placebo-controlled, double-blind, three-period, complete-block, crossover study registered with ClinicalTrials.gov (NCT01311661). After an initial screening visit, patients entered a screening period of 2 weeks to ensure clinical stability. Eligible patients were randomly assigned to receive a three-period treatment sequence comprising 5 μg QD olodaterol (taken in the morning), 2.5 μg BID olodaterol and placebo in a random order, or a three-period treatment sequence comprising 10 μg QD olodaterol (taken in the morning), 5 μg BID olodaterol and placebo in a random order. There were six possible sequences of olodaterol QD, olodaterol BID and placebo for each of the two total daily doses, which ensured that each treatment appeared in each period the same number of times, and each treatment followed every other treatment the same number of times. Patients received each dose regimen for 3 weeks, with a 2-week washout period between regimens (Fig. 1). Treatment was delivered via the Respimat®, with each administration of olodaterol comprising two actuations. All patients were required to take ICS throughout the trial as background medication, as previously instructed by their prescribing physician (BID, QD in the morning or QD in the evening). If administrations coincided, patients were instructed to take the study medications first followed by the ICS.

### Assessments

Spirometry was conducted according to American Thoracic Society and European Respiratory Society recommendations [23]. Identical spirometry equipment was provided to sites for use in clinic measurements. The qualifying pulmonary function test was conducted at screening. Pulmonary function testing (FEV_1_, forced vital capacity [FVC] and PEF) was performed at 1 h and 10 min prior to trial drug inhalation for the morning dose at all visits, ≤3 h post-dose at the start of each 3-week treatment period (weeks 0, 5 and 10 relative to study baseline) and ≤24 h after inhalation (00:30, 01:00, 02:00, 03:00, 04:00, 06:00, 08:00, 10:00, 11:50, 12:30, 13:00, 14:00, 22:00, 23:00, 23:50 h) at the end of each 3-week treatment period (weeks 3, 8 and 13 relative to study baseline), with a period for night sleep. The primary end point was FEV_1_ area under the curve from 0 to 24 h (AUC_0–24_) response at 3 weeks; this was defined as FEV_1_ AUC_0–24_ divided by 24 to report in litres minus the study baseline mean FEV_1_ value. The two key secondary end points were calculated in a similar fashion: FEV_1_ area under the curve from 0 to 12 h (AUC_0–12_) response and FEV_1_ area under the curve from 12 to 24 h (AUC_12–24_) response, all assessed at the end of each 3-week treatment period. Peak values within 24 h post-dose were defined as the maximum available value between the planned time points 00:30 and 23:50 h after last morning trial-drug inhalation (inclusive), while trough values were defined as the mean of the available values at the planned time points 23:00 and 23:50 h. Similar end points were defined for FVC and PEF.

Daily data on asthma symptoms, night-time awakenings, study medication use during treatment period, ICS medication use, amount of rescue medication (salbutamol) use, and morning and evening PEF and FEV_1_ were recorded using an electronic peak flow meter with integral patient diary and reviewed by the investigator at each visit. The Asthma Control Questionnaire [24] was completed at screening and weeks 0, 3, 8, 13 and 15, relative to study baseline.

Adverse events (AEs) and serious AEs were monitored throughout the trial at each patient visit. Vital signs, 12-lead electrocardiogram and laboratory tests were also monitored throughout the study, and reported as AEs if they were not associated with a symptom or a diagnosis already reported as an AE.

### Statistical analysis

A total sample size of 180 randomised patients was planned to provide ≥90 % power to detect a difference in means of 0.10 L between any one of the active treatments and placebo for the primary end point of FEV_1_ AUC_0–24_, assuming a standard deviation for the paired differences of 0.25 L and allowing for approximately 20 % of patients to be non-evaluable.

The full analysis set (FAS) was defined as all patients who received at least one dose of study treatment, for whom a study baseline FEV_1_ value was available and who had at least one post-dose FEV_1_ value recorded at an end-of-treatment visit for at least one crossover period. The FAS was used as the basis for the efficacy analyses, including the primary analysis of the primary end point. A per-protocol set was defined (from which patients with important protocol violations were excluded) and was used to perform a sensitivity analysis on the primary end point.

Results for all patients receiving placebo were pooled for the primary analysis, irrespective of whether they received the lower or higher olodaterol daily dose according to the randomised treatment sequence, to use all available data and increase the precision of the estimates. A *post hoc* sensitivity analysis was also performed on the FAS with placebo split according to treatment sequence (i.e. within each separate complete-block design).

FEV_1_ AUC_0–24_ response was analysed in the FAS using a mixed model for crossover studies with ‘treatment’, ‘period’ and ‘study baseline FEV_1_’ as fixed effects and ‘patient’ as a random effect. The adjusted mean difference between each olodaterol dose/frequency and placebo was calculated using this model, along with the associated *p* values and 95 % confidence intervals (CIs). Secondary end points were also analysed in the FAS using a mixed model for a crossover study, similar to the primary analysis model. The same statistical model specified above was also used for an exploratory analysis undertaken to compare the different doses and frequencies of olodaterol.

Safety end points were summarised descriptively using the treated set (all randomised patients who were dispensed study medication and were documented to have taken at least one dose of investigational treatment).

## Results

### Patient population

**Fig. 2:**
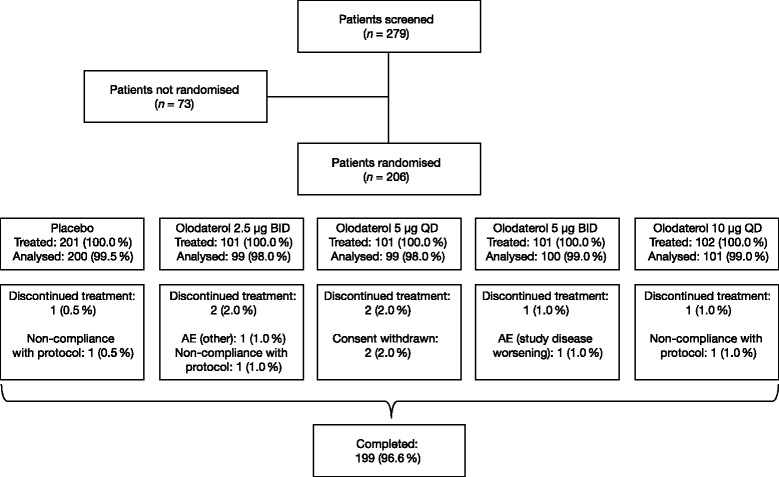
CONSORT diagram illustrating participant flow. Since this was a crossover trial and every patient was supposed to receive three treatments, the total number of patients is not the sum of the number of each patient on each treatment. Of the seven patients who discontinued prematurely, the most frequent reason was non-compliance with the trial protocol (three patients). BID: twice daily; QD: once daily; AE: adverse event

A total of 206 patients at 36 sites in six countries (Austria, Germany, Hungary, Slovakia, Slovenia and the USA) were randomised (Table 1): the population comprised a relatively even proportion of women to men (53 % versus 47 %), most were aged <50 years and the majority had never smoked. Overall, 199 patients (96.6 %) completed the study (Fig. 2).

**Table 1:** Baseline patient demographics (treated set)

	Total (*n* = 206)
Sex, *n* (%)	
Male	97 (47.1)
Female	109 (52.9)
Median (range) age, years	44 (19–69)
Age group, *n* (%)	
<40 years	73 (35.4)
40–50 years	66 (32.0)
51–65 years	60 (29.1)
>65 years	7 (3.4)
Race, *n* (%)	
Caucasian	191 (92.7)
Black/African-American	15 (7.3)
Smoking history, *n* (%)	
Never smoked	144 (69.9)
Ex-smoker^a^	62 (30.1)
Mean FEV_1_, L (standard deviation)	
Pre-bronchodilator^b^	2.433 (0.619)
Post-bronchodilator^c^	2.966 (0.791)

^a^Cigarette smoking history of <10 pack-years and smoking cessation ≥1 year prior to enrolment

^b^10 min before administration of 400 μg salbutamol

^c^10–15 min after administration of 400 μg salbutamol, *n* = 205

*FEV*_*1*_ forced expiratory volume in 1 s

### Efficacy

#### Lung function

**Fig. 3:**
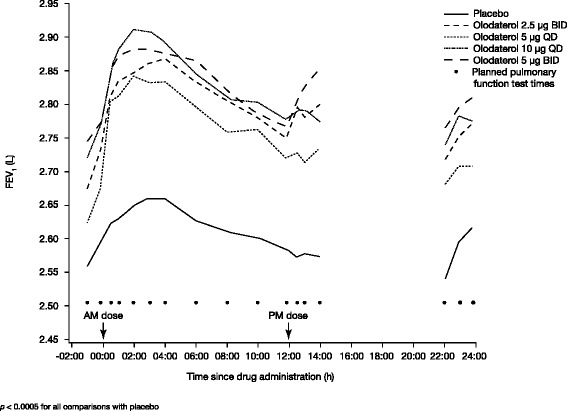
Adjusted mean FEV_1_: individual time points from 0 to 24 h at 3 weeks. Analysis with imputation, full analysis set. BID: twice daily; QD: once daily; FEV_1_: forced expiratory volume in 1 s

Highly statistically significant improvements in FEV_1_ AUC_0–24_ response were observed with all doses and dose frequencies of olodaterol compared to placebo at week 3 (*p* < 0.0001) (Table 2), with similar results demonstrated for individual FEV_1_ values at all individual time points (*p* < 0.0005) (Fig. 3).

**Table 2:** Adjusted mean FEV_1_ AUC_0–24_, AUC_0–12_ and AUC_12–24_ responses at 3 weeks

				Difference from placebo, L		
End point	Treatment, μg	*n*	Mean FEV_1_ AUC response, L (SE)	Mean (SE)	95 % CI	*p* value
FEV_1_ AUC_0–24_ response	Placebo	200	0.022 (0.020)			
	Olodaterol 2.5 BID	99	0.213 (0.024)	0.191 (0.020)	0.152, 0.229	<0.0001
	Olodaterol 5 QD	99	0.173 (0.024)	0.150 (0.020)	0.111, 0.189	<0.0001
	Olodaterol 5 BID	100	0.250 (0.024)	0.228 (0.020)	0.190, 0.266	<0.0001
	Olodaterol 10 QD	101	0.231 (0.024)	0.209 (0.020)	0.170, 0.247	<0.0001
FEV_1_ AUC_0–12_ response	Placebo	200	0.052 (0.020)			
	Olodaterol 2.5 BID	99	0.242 (0.024)	0.190 (0.020)	0.150, 0.229	<0.0001
	Olodaterol 5 QD	99	0.212 (0.024)	0.160 (0.020)	0.121, 0.199	<0.0001
	Olodaterol 5 BID	100	0.266 (0.024)	0.214 (0.020)	0.175, 0.253	<0.0001
	Olodaterol 10 QD	101	0.272 (0.024)	0.219 (0.020)	0.181, 0.258	<0.0001
FEV_1_ AUC_12–24_ response	Placebo	201	-0.010 (0.020)			
	Olodaterol 2.5 BID	99	0.186 (0.025)	0.196 (0.022)	0.153, 0.238	<0.0001
	Olodaterol 5 QD	99	0.135 (0.025)	0.144 (0.022)	0.102, 0.187	<0.0001
	Olodaterol 5 BID	100	0.233 (0.025)	0.242 (0.022)	0.200, 0.285	<0.0001
	Olodaterol 10 QD	101	0.189 (0.025)	0.198 (0.022)	0.156, 0.241	<0.0001

Common study baseline mean (SE): 2.571 (0.054) for all end points

*FEV*_*1*_ forced expiratory volume in 1 s, *AUC*_*0–24*_ area under the curve from 0 to 24 h, *AUC*_*0–12*_ area under the curve from 0 to 12 h, *AUC*_*12–24*_ area under the curve from 12 to 24 h, *SE* standard error, *CI* confidence interval, *BID* twice daily, *QD* once daily

For the within-crossover group comparisons of the same total daily dose, the mean FEV_1_ AUC_0–24_ response for treatment with olodaterol 2.5 μg BID (0.213 L) was greater than for olodaterol 5 μg QD (0.173 L; *p* = 0.0465; adjusted mean difference for 5 μg QD versus 2.5 μg BID -0.040 L [95 % CI -0.080, -0.001]). There was no evidence of a difference between treatment with olodaterol 10 μg QD and 5 μg BID (*p* = 0.3388; adjusted mean difference -0.019 L [95 % CI -0.059, 0.020]).

In the secondary, exploratory analysis of two different total daily doses (i.e. parallel-group comparison), there was no significant difference between mean FEV_1_ AUC_0–24_ response with olodaterol 5 μg BID and 2.5 μg BID (*p* = 0.1631; adjusted mean difference 0.037 L [95 % CI -0.015, 0.090]), while olodaterol 10 μg QD provided a greater mean FEV_1_ AUC_0–24_ response than olodaterol 5 μg QD (*p* = 0.0289; adjusted mean difference 0.059 L [95 % CI 0.006, 0.111]). Mean FEV_1_ AUC_0–24_ response was higher with olodaterol 5 μg BID compared to olodaterol 5 μg QD (*p* = 0.0038; adjusted mean difference 0.078 L [95 % CI 0.025, 0.130]).

The *post hoc* sensitivity analyses performed with placebo split according to treatment sequence were consistent with the primary analysis (*p* < 0.0001 for all comparisons with placebo) and gave similar estimates for treatment effect.

Analysis of the key secondary end points of FEV_1_ AUC_0–12_ and AUC_12–24_ responses supported the outcomes of the primary end point and provided additional information from within the 24-h measurement period for olodaterol QD and BID, indicating increased adjusted mean FEV_1_ AUC_12–24_ response for BID versus QD of the same daily dose, as would be expected (Table 2). Adjusted mean FEV_1_ at individual time points are shown in Additional file 1: Table S1.

**Fig. 4:**
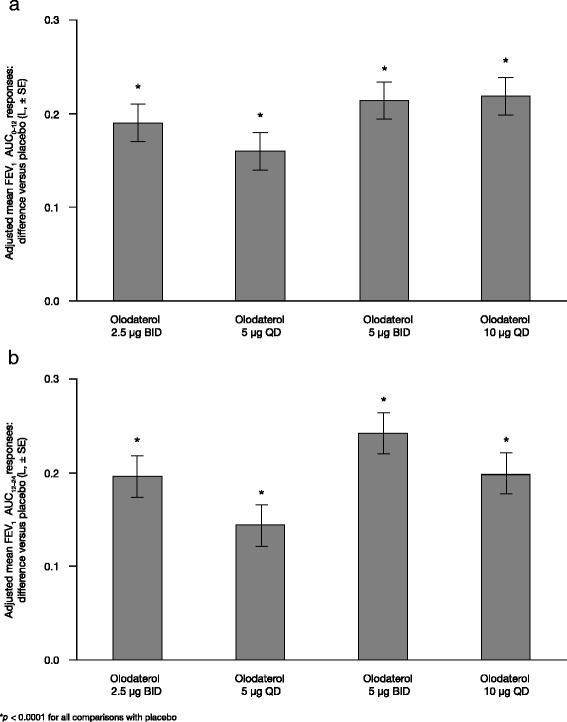
Difference versus placebo at 3 weeks of adjusted mean FEV_1_ AUC_0–12_ (**a**) and AUC_12–24_ (**b**). FEV_1_: forced expiratory volume in 1 s; AUC_0–12_: area under the curve from 0 to 12 h; SE: standard error; BID: twice daily; QD: once daily; AUC_12–24_: area under the curve from 12 to 24 h

Increased adjusted mean FEV_1_ AUC_0–12_ response with 2.5 μg BID versus 5 μg QD, but not with 5 μg BID versus 10 μg QD, was identified (Fig. 4a; Table 2). The adjusted mean difference from placebo in FEV_1_ AUC_0–12_ response ranged from 0.160 L (5 μg olodaterol QD; 95 % CI 0.121, 0.199) to 0.219 L (10 μg olodaterol QD; 95 % CI 0.181, 0.258), while results for FEV_1_ AUC_12–24_ response ranged from 0.144 L (5 μg olodaterol QD; 95 % CI 0.102, 0.187) to 0.242 L (5 μg olodaterol BID; 95 % CI 0.200, 0.285) (Fig. 4b). For both end points, an increase in adjusted mean difference from placebo was observed with an increasing total daily dose of olodaterol at both dosing frequencies (*p* < 0.0001 for all comparisons with placebo).

The analysis of FEV_1_ at all individual time points assessed at each visit after 3 weeks of treatment showed strong evidence of efficacy (*p* < 0.0005 for comparisons with placebo) with all olodaterol doses and dosing frequencies at all post-dosing points on the 24-h curve.

**Fig. 5:**
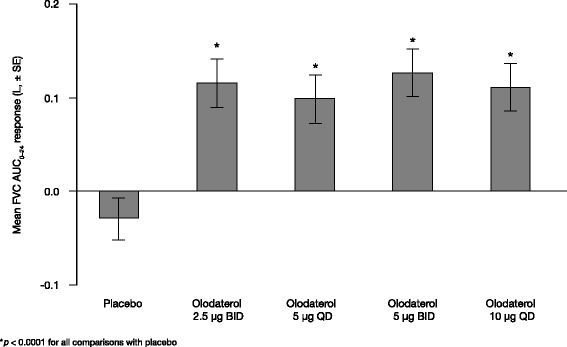
Adjusted mean FVC AUC_0–24_ at 3 weeks. FVC: forced vital capacity; AUC_0–24_: area under the curve from 0 to 24 h; SE: standard error; BID: twice daily; QD: once daily

Assessment of mean FVC AUC_0–24_ response supported the results of the primary end point (Fig. 5), as did peak and trough FEV_1_ and FVC responses (Table 3 and see Additional file 1: Table S2) and PEF AUC_0–24_ (see Additional file 1: Table S3), peak and trough responses (data not shown).

**Table 3:** Adjusted mean peak and trough FEV_1_ and FVC responses at 3 weeks

				Difference from placebo, L		
End point	Treatment, μg	*n*	Mean FEV_1_/FVC response, L (SE)	Mean (SE)	95 % CI	*p* value
FEV_1_ peak response	Placebo	201	0.227 (0.021)			
	Olodaterol 2.5 BID	99	0.410 (0.027)	0.183 (0.023)	0.138, 0.228	<0.0001
	Olodaterol 5 QD	99	0.380 (0.027)	0.153 (0.023)	0.108, 0.198	<0.0001
	Olodaterol 5 BID	100	0.449 (0.027)	0.222 (0.023)	0.177, 0.267	<0.0001
	Olodaterol 10 QD	101	0.437 (0.026)	0.210 (0.023)	0.165, 0.255	<0.0001
FEV_1_ trough response	Placebo	201	0.033 (0.022)			
	Olodaterol 2.5 BID	99	0.189 (0.027)	0.156 (0.024)	0.109, 0.203	<0.0001
	Olodaterol 5 QD	99	0.134 (0.027)	0.101 (0.024)	0.054, 0.148	<0.0001
	Olodaterol 5 BID	100	0.229 (0.027)	0.196 (0.024)	0.149, 0.243	<0.0001
	Olodaterol 10 QD	101	0.205 (0.027)	0.172 (0.024)	0.125, 0.219	<0.0001
FVC peak response	Placebo	201	0.246 (0.024)			
	Olodaterol 2.5 BID	99	0.382 (0.030)	0.136 (0.026)	0.084, 0.187	<0.0001
	Olodaterol 5 QD	99	0.371 (0.030)	0.124 (0.026)	0.073, 0.176	<0.0001
	Olodaterol 5 BID	100	0.390 (0.030)	0.144 (0.026)	0.093, 0.195	<0.0001
	Olodaterol 10 QD	101	0.373 (0.029)	0.126 (0.026)	0.075, 0.177	<0.0001
FVC trough response	Placebo	201	-0.013 (0.024)			
	Olodaterol 2.5 BID	99	0.096 (0.031)	0.110 (0.028)	0.055, 0.165	0.0001
	Olodaterol 5 QD	99	0.079 (0.031)	0.092 (0.028)	0.037, 0.147	0.0010
	Olodaterol 5 BID	100	0.105 (0.031)	0.118 (0.028)	0.063, 0.172	<0.0001
	Olodaterol 10 QD	101	0.098 (0.031)	0.111 (0.028)	0.057, 0.166	<0.0001

Common study baseline mean (SE): 2.571 (0.054) for FEV_1_ and 3.849 (0.073) for FVC

*FEV*_*1*_ forced expiratory volume in 1 s, *FVC* forced vital capacity, *SE* standard error, *CI* confidence interval, *BID* twice daily, *QD* once daily

#### Other efficacy variables

All doses and dosing frequencies of olodaterol provided statistically significant improvements in mean morning and evening PEF (from patient diaries) and total Asthma Control Questionnaire scores at 3 weeks compared to placebo (*p* < 0.0001 for all comparisons with placebo) (see Additional file 1: Table S4). No relevant differences were observed between different doses and regimens.

#### Safety

The overall proportion of patients who reported at least one AE while on treatment was 35.4 %. Incidence of AEs was highest for 5 μg BID olodaterol and lowest for 10 μg QD, although, in general, AEs were in a similar range (placebo 16.4 %; olodaterol 2.5 μg BID and 5 μg QD 14.9 %; 5 μg BID 18.8 %; 10 μg QD 12.7 %) (Table 4). Overall incidence of AEs by sex was 34.0 % for men and 36.7 % for women, with the largest difference reported with placebo treatment (11.7 % of men versus 20.6 % of women reporting any AE). Serious AEs were reported for four patients in total (1.9 %): two patients (1.0 %) for placebo, one patient in the washout period 4 days after discontinuing 5 μg BID olodaterol treatment and one patient 9 days after starting olodaterol 2.5 μg BID. None was considered to be treatment-related.

**Table 4:** Summary of AEs

	Placebo, *n* (%)	Olodaterol 2.5 μg BID, *n* (%)	Olodaterol 5 μg QD, *n* (%)	Olodaterol 5 μg BID, *n* (%)	Olodaterol 10 μg QD, *n* (%)	Total, *n* (%)
All AEs	33 (16.4)	15 (14.9)	15 (14.9)	19 (18.8)	13 (12.7)	73 (35.4)
Treatment-related AEs	2 (1.0)	0	1 (1.0)	3 (3.0)	1 (1.0)	7 (3.4)
AEs leading to discontinuation	0	1 (1.0)	0	1 (1.0)	0	2 (1.0)
Serious AEs	2 (1.0)	1 (1.0)	0	1 (1.0)	0	4 (1.9)
Specific AEs with an incidence >1 %						
Asthma	4 (2.0)	1 (1.0)	1 (1.0)	2 (2.0)	1 (1.0)	9 (4.4)
Injury, poisoning and procedural complications	2 (1.0)	3 (3.0)	0	3 (3.0)	1 (1.0)	9 (4.4)
Headache	5 (2.5)	1 (1.0)	2 (2.0)	0	3 (2.9)	9 (4.4)
Sinusitis	1 (0.5)	0	0	0	4 (3.9)	5 (2.4)
Nasopharyngitis	3 (1.5)	0	0	1 (1.0)	0	4 (1.9)
Musculoskeletal and connective tissue disorders	0	2 (2.0)	1 (1.0)	1 (1.0)	0	4 (1.9)
Upper respiratory tract infection	3 (1.5)	1 (1.0)	0	1 (1.0)	0	4 (1.9)
Cystitis	1 (0.5)	0	0	2 (2.0)	0	2 (1.0)
Eye disorders	1 (0.5)	0	0	2 (2.0)	0	2 (1.0)
Rhinitis allergic	0	2 (2.0)	0	0	0	2 (1.0)
Skin and cutaneous tissue disorders	0	0	2 (2.0)	0	0	2 (1.0)

The preferred term ‘asthma’ summarises several lowest level terms; in the majority of cases, the reported level term was ‘exacerbation of asthma’

*AE* adverse event, *BID* twice daily, *QD* once daily

Overall, the most frequently reported AEs were infections and infestations (14.1 %), with sinusitis the most frequently reported AE within this category (2.4 % overall). Respiratory, thoracic and mediastinal disorders were the second most frequently reported AE (9.2 %), with the highest rate reported for placebo, at 4.5 %. Within this category, the most common AE reported was asthma (4.4 % overall) with the highest reporting frequency of 2.0 % for placebo and olodaterol 5 μg BID (Table 4).

A total of seven patients (3.4 %) were considered to have had AEs related to study drug by the investigators. Two patients were receiving placebo (1.0 %; headache and cough); two patients who had cough were receiving olodaterol (one receiving 5 μg QD, one receiving 10 μg QD; 1.0 % in both instances). Insomnia, palpitations and asthma were reported by three patients taking 5 μg BID olodaterol (3.0 %). No notable findings were reported via assessment of laboratory parameters, vital signs and electrocardiogram readings.

## Discussion

The results of this study add support to the growing evidence base that olodaterol delivered by Respimat^®^ QD provides effective 24-h bronchodilation in both COPD and asthma [9–11, 13–16, 25]. This study was designed to assess the efficacy of different daily doses and dose frequencies of olodaterol versus placebo, and between different doses and frequencies of olodaterol in an exploratory fashion, in patients with moderate to severe persistent asthma after 3 weeks of treatment, thereby providing additional information on the efficacy of QD versus BID dosing.

Both total daily doses and all dosing frequencies of olodaterol tested provided highly significant improvements versus placebo in efficacy variables based on pulmonary function tests, albeit with different 24-h bronchodilatory profiles. Differences were seen between doses and posologies of olodaterol, with a greater mean FEV_1_ AUC_0–24_ response for 2.5 μg BID than 5 μg olodaterol QD in the morning but no statistically significant difference was observed between olodaterol 5 μg BID and olodaterol 10 μg QD in the morning.

As expected, the second daily dose in BID schedules gave increased mean FEV_1_ AUC_12–24_ response versus QD dosing at the same daily dose after 3 weeks of treatment. However, while a higher mean FEV_1_ AUC_0–12_ response might be anticipated for the QD dosing (as was observed for mean area under the curve from 0 to 3 h response at the start of the 3-week treatment period [data not shown]), this was not observed.

Pre-dose morning FEV_1_ measurements at -1 h and -10 min in this study were numerically higher for both the BID posologies versus QD posologies but notably for 2.5 μg olodaterol BID, hinting at a residual effect; however, further investigation would be required to fully evaluate this potential effect. An inspection of the placebo versus olodaterol time profiles confirms that the reported circadian influence on lung function [26] is not abolished but rather the level of bronchodilation is increased by olodaterol treatment.

Interpretation of the exploratory data in the present study should, however, take into consideration that the study was designed to compare different doses and dosing frequencies of olodaterol with placebo. Consequently, it was not powered to examine statistical differences between active treatments, nor was there any adjustment made for such multiple treatment comparisons. As individual patients only received one total daily dose, inter-patient variability also contributed to increasing uncertainty in the estimation of effect-size comparisons of different daily doses in the parallel arms (wider CIs). Therefore, as is generally true for Phase II studies, any interpretation of the comparisons between different doses and dose frequencies of olodaterol should be undertaken with caution.

Although this study provided some information on the difference in efficacy of different posologies of the same daily dose of olodaterol on top of ICS, along with demonstrating adequate short-term safety, further, longer-term research would be required to fully examine the optimum olodaterol dose regimen in patients with asthma, possibly investigating patient type, combination treatment and the effect of variations in the timing of QD dosing (e.g. morning versus evening dosing).

The lung function efficacy of olodaterol versus placebo has also been demonstrated by other Phase II olodaterol trials carried out in asthma [20, 21]. O’Byrne *et al*. reported results of their placebo-controlled, parallel-group study in 426 patients (NCT00467740) at the American Thoracic Society meeting in 2012 [20] and demonstrated dose-dependent improvements in lung function with QD olodaterol, although dose ordering was not consistently shown for each of the individual efficacy end points. While the primary end point of trough FEV_1_ demonstrated numerical improvement for all doses, including 5 and 10 μg olodaterol, only the improvements observed in patients receiving 20 μg olodaterol QD were statistically significant. Evidence of dose ordering was observed in the pre-dose morning PEF response, with dose-related improvements also observed on the Asthma Control Questionnaire [20]. Due to lack of dose ordering seen for the primary end point in this study, Beeh *et al*. conducted a similar study, but using an incomplete-block crossover design, in 198 patients (NCT01013753) [21]. This study also demonstrated highly statistically significant improvements in FEV_1_ and PEF response with all doses of olodaterol examined and clear evidence of dose ordering [21].

The results of our study provide further evidence for the dose ordering seen between 5 and 10 μg total daily doses; the parallel-group comparison of 5 μg QD versus 10 μg QD for several FEV_1_ response assessments demonstrates a dose response with increasing dose, and further evidence for this relationship is supplied by the comparison of 2.5 μg BID versus 5 μg BID. Indeed, a dose response is also observed when doubling the dose from 5 μg QD to 5 μg BID. When considered as a whole, the weight of the current evidence from several olodaterol trials in asthma indicates a relevant dose-response relationship between a total daily dose of 5 and 10 μg olodaterol.

Increases in FVC observed with all doses of olodaterol were lower than the increases seen with FEV_1_. This may indicate the effect of olodaterol on peripheral airways, as air trapping is often a feature of small-airway disease in patients with severe asthma [27]. Indeed, bronchodilation using high doses of albuterol has been demonstrated to decrease residual volume:total lung capacity ratio while increasing FVC in parallel, with improvements in FVC partially responsible for increase in FEV_1_ [27].

A series of clinical studies has examined the dose dependence and posology of olodaterol bronchodilatory efficacy in COPD. A single-dose study [10] and a study of 4-week QD treatment [11] have demonstrated 24-h bronchodilation in COPD. In order to investigate dosing frequency, a complete-block crossover study (NCT00846768) investigated the 24-h bronchodilator profile of different olodaterol regimens in 47 patients with COPD [12]. Although it did not include a placebo treatment, the study demonstrated statistically significant improvements versus baseline for all doses and dose regimens: 5 and 10 μg QD provided near-identical bronchodilation over a 24-h period, while 5 μg QD demonstrated an improved bronchodilation profile versus 2 μg BID [12], thus providing support for the further investigation of 5 and 10 μg QD in COPD [13–16, 28]. Olodaterol 5 μg QD is now approved in several countries for the treatment of COPD. Together with the current study, these data suggest that olodaterol provides effective 24-h bronchodilation in both asthma and COPD. The data in COPD provide a clear rationale for QD schedules; the current data in asthma are limited and less clear, although all doses and schedules provided effective 24-h bronchodilation.

Thorough investigation of new LABAs in asthma is of particular relevance due to the concerns over safety of this class of agent. LABA monotherapy in asthma is contraindicated to minimise the risk of serious exacerbations of asthma and asthma-associated deaths [17]; it is for this reason that patients in the present study were required to continue taking ICS as background medication. To ensure a full picture of the safety of new LABAs, the US Food and Drug Administration also considered asthma safety data when approving the LABA indacaterol in COPD at 75 μg QD (a lower dose than the EU) [29]. With this in mind, the current study demonstrated an acceptable short-term safety profile for olodaterol in asthma on top of maintenance ICS. Incidence of AEs was similar between olodaterol and placebo, and ranged from 12.7 to 18.8 %. All three studies in asthma conducted to date have shown olodaterol to have consistent short-term safety and tolerability profiles, and have raised no safety concerns [20, 21], in line with previous studies in COPD [11, 12].

In conclusion, in this posology study in patients with moderate to severe asthma, while resulting in some differences in 24-h bronchodilation profile, all daily doses (5 and 10 μg) and dose frequencies (2.5 μg BID, 5 μg QD, 5 μg BID, 10 μg QD) of olodaterol demonstrated 24-h bronchodilation superior to placebo in patients with asthma on a background of inhaled steroids. These data should be interpreted with caution, as further studies would be required in asthma before making clinical decisions on optimal dosing and posology. No safety concerns were identified and the short-term safety and tolerability profiles of olodaterol were similar to those seen in previous trials. Further, long-term research would be required in order to fully examine the long-term safety and optimum dose regimen of olodaterol in patients with asthma.

## Additional file

Additional file 1: Table S1. Adjusted mean FEV_1_ and comparison with placebo at individual time points at 3 weeks. **Table S2.** Adjusted mean FVC AUC_0–24_ response and comparison with placebo at 3 weeks. **Table S3.** Adjusted mean PEF AUC_0–24_ response and comparison with placebo at 3 weeks. **Table S4.** Overall adjusted mean PEF and total Asthma Control Questionnaire score after 3 weeks.

## Abbreviations

AE: Adverse event
AUC_0–12_: Area under the curve from 0 to 12 h
AUC_0–24_: Area under the curve from 0 to 24 h
AUC_12–24_: Area under the curve from 12 to 24 h
BID: Twice daily
CI: Confidence interval
COPD: Chronic obstructive pulmonary disease
FAS: Full analysis set
FEV_1_: Forced expiratory volume in 1 s
FVC: Forced vital capacity
ICS: Inhaled corticosteroids
LABA: Long-acting β_2_-agonist
PEF: Peak expiratory flow
QD: Once daily
