# Supplementary material for: Randomised, double-blind, placebo-controlled crossover study to investigate different dosing regimens of olodaterol delivered via Respimat® in patients with moderate to severe persistent asthma
Source: Respir Res. 2015 Jul 16;16(1):87. doi: 10.1186/s12931-015-0243-1 (PMC4509767; doi:10.1186/s12931-015-0243-1)
Supplement: Additional file 1: Table S1. — Adjusted mean FEV1 and comparison with placebo at individual time points at 3 weeks. Table S2. Adjusted mean FVC AUC0–24 response and comparison with placebo at 3 weeks. Table S3. Adjusted mean PEF AUC0–24 response and comparison with placebo at 3 weeks. Table S4. Overall adjusted mean PEF and total Asthma Control Questionnaire score after 3 weeks. [file 12931_2015_243_MOESM1_ESM.doc]

**Additional Table S1 Adjusted mean FEV_1_ and comparison with placebo at individual time points at 3 weeks**

|  |  |  |  | **Difference from placebo, L** | | |
| --- | --- | --- | --- | --- | --- | --- |
| **Planned time, hour** | **Treatment, µg** | **n** | **Mean FEV_1_, L (SE)** | **Mean (SE)** | **95% CI** | **p value** |
| -01:00 | Placebo | 200 | 2.559 (0.021) |  |  |  |
|  | Olodaterol 2.5BID | 99 | 2.677 (0.026) | 0.117 (0.024) | 0.071, 0.164 | < 0.0001 |
|  | Olodaterol 5 QD | 99 | 2.625 (0.026) | 0.066 (0.024) | 0.019, 0.113 | 0.0060 |
|  | Olodaterol 5 BID | 100 | 2.744 (0.026) | 0.185 (0.024) | 0.138, 0.231 | < 0.0001 |
|  | Olodaterol 10 QD | 101 | 2.719 (0.026) | 0.160 (0.024) | 0.113, 0.206 | < 0.0001 |
| -00:10 | Placebo | 200 | 2.593 (0.020) |  |  |  |
|  | Olodaterol 2.5 BID | 99 | 2.730 (0.026) | 0.137 (0.023) | 0.093, 0.182 | < 0.0001 |
|  | Olodaterol 5 QD | 99 | 2.676 (0.026) | 0.083 (0.023) | 0.038, 0.128 | 0.0003 |
|  | Olodaterol 5 BID | 100 | 2.778 (0.026) | 0.185 (0.023) | 0.141, 0.230 | < 0.0001 |
|  | Olodaterol 10 QD | 101 | 2.766 (0.025) | 0.173 (0.023) | 0.129, 0.218 | < 0.0001 |
| 00:30 | Placebo | 201 | 2.623 (0.020) |  |  |  |
|  | Olodaterol 2.5 BID | 99 | 2.812 (0.024) | 0.189 (0.021) | 0.147, 0.231 | < 0.0001 |
|  | Olodaterol 5 QD | 99 | 2.804 (0.024) | 0.181 (0.021) | 0.139, 0.222 | < 0.0001 |
|  | Olodaterol 5 BID | 100 | 2.850 (0.024) | 0.226 (0.021) | 0.185, 0.268 | < 0.0001 |
|  | Olodaterol 10 QD | 101 | 2.855 (0.024) | 0.232 (0.021) | 0.190, 0.274 | < 0.0001 |
| 01:00 | Placebo | 201 | 2.629 (0.021) |  |  |  |
|  | Olodaterol 2.5 BID | 99 | 2.833 (0.025) | 0.204 (0.021) | 0.162, 0.246 | < 0.0001 |
|  | Olodaterol 5 QD | 99 | 2.812 (0.025) | 0.183 (0.021) | 0.140, 0.225 | < 0.0001 |
|  | Olodaterol 5 BID | 100 | 2.873 (0.025) | 0.244 (0.021) | 0.202, 0.286 | < 0.0001 |
|  | Olodaterol 10 QD | 101 | 2.883 (0.025) | 0.254 (0.021) | 0.212, 0.296 | < 0.0001 |
| 02:00 | Placebo | 201 | 2.651 (0.021) |  |  |  |
|  | Olodaterol 2.5 BID | 99 | 2.846 (0.026) | 0.195 (0.022) | 0.152, 0.239 | < 0.0001 |
|  | Olodaterol 5 QD | 99 | 2.842 (0.026) | 0.191 (0.022) | 0.147, 0.234 | < 0.0001 |
|  | Olodaterol 5 BID | 100 | 2.881 (0.026) | 0.230 (0.022) | 0.187, 0.274 | < 0.0001 |
|  | Olodaterol 10 QD | 101 | 2.912 (0.026) | 0.261 (0.022) | 0.218, 0.305 | < 0.0001 |
| 03:00 | Placebo | 201 | 2.661 (0.021) |  |  |  |
|  | Olodaterol 2.5 BID | 99 | 2.862 (0.025) | 0.201 (0.022) | 0.159, 0.244 | < 0.0001 |
|  | Olodaterol 5 QD | 99 | 2.832 (0.025) | 0.171 (0.022) | 0.128, 0.214 | < 0.0001 |
|  | Olodaterol 5 BID | 100 | 2.881 (0.025) | 0.220 (0.022) | 0.178, 0.263 | < 0.0001 |
|  | Olodaterol 10 QD | 101 | 2.908 (0.025) | 0.247 (0.022) | 0.205, 0.290 | < 0.0001 |
| 04:00 | Placebo | 201 | 2.660 (0.021) |  |  |  |
|  | Olodaterol 2.5 BID | 99 | 2.868 (0.026) | 0.208 (0.023) | 0.163, 0.254 | < 0.0001 |
|  | Olodaterol 5 QD | 99 | 2.833 (0.026) | 0.174 (0.023) | 0.128, 0.220 | < 0.0001 |
|  | Olodaterol 5 BID | 100 | 2.877 (0.026) | 0.218 (0.023) | 0.172, 0.263 | < 0.0001 |
|  | Olodaterol 10 QD | 101 | 2.895 (0.026) | 0.235 (0.023) | 0.190, 0.281 | < 0.0001 |
| 06:00 | Placebo | 201 | 2.627 (0.021) |  |  |  |
|  | Olodaterol 2.5 BID | 99 | 2.834 (0.028) | 0.206 (0.026) | 0.155, 0.258 | < 0.0001 |
|  | Olodaterol 5 QD | 99 | 2.795 (0.028) | 0.168 (0.026) | 0.117, 0.220 | < 0.0001 |
|  | Olodaterol 5 BID | 100 | 2.864 (0.028) | 0.237 (0.026) | 0.186, 0.288 | < 0.0001 |
|  | Olodaterol 10 QD | 101 | 2.847 (0.028) | 0.219 (0.026) | 0.168, 0.271 | < 0.0001 |
| 08:00 | Placebo | 201 | 2.611 (0.022) |  |  |  |
|  | Olodaterol 2.5 BID | 99 | 2.806 (0.028) | 0.195 (0.025) | 0.145, 0.245 | < 0.0001 |
|  | Olodaterol 5 QD | 99 | 2.760 (0.028) | 0.149 (0.025) | 0.099, 0.199 | < 0.0001 |
|  | Olodaterol 5 BID | 100 | 2.821 (0.028) | 0.210 (0.025) | 0.160, 0.260 | < 0.0001 |
|  | Olodaterol 10 QD | 101 | 2.808 (0.027) | 0.197 (0.025) | 0.147, 0.247 | < 0.0001 |
| 10:00 | Placebo | 201 | 2.602 (0.022) |  |  |  |
|  | Olodaterol 2.5 BID | 99 | 2.781 (0.028) | 0.179 (0.024) | 0.131, 0.227 | < 0.0001 |
|  | Olodaterol 5 QD | 99 | 2.762 (0.028) | 0.161 (0.024) | 0.113, 0.208 | < 0.0001 |
|  | Olodaterol 5 BID | 100 | 2.786 (0.028) | 0.185 (0.024) | 0.137, 0.232 | < 0.0001 |
|  | Olodaterol 10 QD | 101 | 2.803 (0.028) | 0.201 (0.024) | 0.154, 0.249 | < 0.0001 |
| 11:50 | Placebo | 201 | 2.584 (0.023) |  |  |  |
|  | Olodaterol 2.5 BID | 99 | 2.748 (0.029) | 0.165 (0.026) | 0.114, 0.216 | < 0.0001 |
|  | Olodaterol 5 QD | 99 | 2.720 (0.029) | 0.137 (0.026) | 0.086, 0.188 | < 0.0001 |
|  | Olodaterol 5 BID | 100 | 2.767 (0.028) | 0.183 (0.026) | 0.133, 0.234 | < 0.0001 |
|  | Olodaterol 10 QD | 101 | 2.776 (0.028) | 0.193 (0.026) | 0.142, 0.243 | < 0.0001 |
| 12:30 | Placebo | 201 | 2.573 (0.021) |  |  |  |
|  | Olodaterol 2.5 BID | 99 | 2.795 (0.027) | 0.221 (0.025) | 0.172, 0.270 | < 0.0001 |
|  | Olodaterol 5 QD | 99 | 2.727 (0.027) | 0.154 (0.025) | 0.105, 0.203 | < 0.0001 |
|  | Olodaterol 5 BID | 100 | 2.807 (0.027) | 0.234 (0.025) | 0.185, 0.282 | < 0.0001 |
|  | Olodaterol 10 QD | 101 | 2.794 (0.027) | 0.220 (0.025) | 0.172, 0.269 | < 0.0001 |
| 13:00 | Placebo | 201 | 2.577 (0.022) |  |  |  |
|  | Olodaterol 2.5 BID | 99 | 2.780 (0.027) | 0.203 (0.025) | 0.154, 0.252 | < 0.0001 |
|  | Olodaterol 5 QD | 99 | 2.715 (0.027) | 0.138 (0.025) | 0.089, 0.186 | < 0.0001 |
|  | Olodaterol 5 BID | 100 | 2.827 (0.027) | 0.250 (0.025) | 0.201, 0.298 | < 0.0001 |
|  | Olodaterol 10 QD | 101 | 2.791 (0.027) | 0.213 (0.025) | 0.165, 0.262 | < 0.0001 |
| 14:00 | Placebo | 201 | 2.574 (0.021) |  |  |  |
|  | Olodaterol 2.5 BID | 99 | 2.799 (0.027) | 0.226 (0.025) | 0.176, 0.276 | < 0.0001 |
|  | Olodaterol 5 QD | 99 | 2.736 (0.027) | 0.162 (0.025) | 0.113, 0.212 | < 0.0001 |
|  | Olodaterol 5 BID | 100 | 2.851 (0.027) | 0.277 (0.025) | 0.228, 0.327 | < 0.0001 |
|  | Olodaterol 10 QD | 101 | 2.774 (0.027) | 0.201 (0.025) | 0.151, 0.250 | < 0.0001 |
| 22:00 | Placebo | 201 | 2.538 (0.023) |  |  |  |
|  | Olodaterol 2.5 BID | 99 | 2.716 (0.028) | 0.178 (0.025) | 0.129, 0.226 | < 0.0001 |
|  | Olodaterol 5 QD | 99 | 2.679 (0.028) | 0.140 (0.025) | 0.092, 0.189 | < 0.0001 |
|  | Olodaterol 5 BID | 100 | 2.762 (0.028) | 0.224 (0.025) | 0.176, 0.272 | < 0.0001 |
|  | Olodaterol 10 QD | 101 | 2.736 (0.028) | 0.197 (0.024) | 0.149, 0.246 | < 0.0001 |
| 23:00 | Placebo | 201 | 2.595 (0.022) |  |  |  |
|  | Olodaterol 2.5 BID | 99 | 2.752 (0.028) | 0.157 (0.026) | 0.107, 0.208 | < 0.0001 |
|  | Olodaterol 5 QD | 99 | 2.708 (0.028) | 0.112 (0.026) | 0.062, 0.163 | < 0.0001 |
|  | Olodaterol 5 BID | 100 | 2.797 (0.028) | 0.202 (0.026) | 0.151, 0.252 | < 0.0001 |
|  | Olodaterol 10 QD | 101 | 2.782 (0.028) | 0.186 (0.026) | 0.136, 0.236 | < 0.0001 |
| 23:50 | Placebo | 201 | 2.618 (0.023) |  |  |  |
|  | Olodaterol 2.5 BID | 99 | 2.773 (0.028) | 0.155 (0.025) | 0.107, 0.204 | < 0.0001 |
|  | Olodaterol 5 QD | 99 | 2.708 (0.028) | 0.090 (0.025) | 0.041, 0.138 | 0.0003 |
|  | Olodaterol 5 BID | 100 | 2.809 (0.028) | 0.191 (0.025) | 0.143, 0.239 | < 0.0001 |
|  | Olodaterol 10 QD | 101 | 2.775 (0.028) | 0.157 (0.025) | 0.109, 0.206 | < 0.0001 |

FEV_1_: forced expiratory volume in 1 second; SE: standard error; CI: confidence interval; BID: twice daily; QD: once daily.

**Additional Table S2 Adjusted mean FVC AUC_0–24_ response and comparison with placebo at 3 weeks**

|  |  |  |  | **Difference from placebo, L** | | |
| --- | --- | --- | --- | --- | --- | --- |
| **End point** | **Treatment, µg** | **n** | **Mean FVC** **AUC response, L (SE)** | **Mean (SE)** | **95% CI** | **p value** |
| FVC AUC_0–24_ response | Placebo | 200 | -0.029 (0.021) |  |  |  |
|  | Olodaterol 2.5 BID | 99 | 0.116 (0.026) | 0.145 (0.022) | 0.102, 0.188 | < 0.0001 |
|  | Olodaterol 5 QD | 99 | 0.099 (0.026) | 0.128 (0.022) | 0.085, 0.171 | < 0.0001 |
|  | Olodaterol 5 BID | 100 | 0.127 (0.026) | 0.156 (0.022) | 0.113, 0.198 | < 0.0001 |
|  | Olodaterol 10 QD | 101 | 0.111 (0.026) | 0.140 (0.022) | 0.098, 0.182 | < 0.0001 |

Common study baseline mean (SE): 3.849 (0.073).
FVC: forced vital capacity; AUC_0–24_: area under the curve from 0–24 hours; SE: standard error; CI: confidence interval; BID: twice daily; QD: once daily.

**Additional Table S3 Adjusted mean PEF AUC_0–24_ response and comparison with placebo at 3 weeks**

|  |  |  |  | **Difference from placebo, L/s** | | |
| --- | --- | --- | --- | --- | --- | --- |
| **End point** | **Treatment, µg** | **n** | **Mean PEF** **AUC response, L/s (SE)** | **Mean (SE)** | **95% CI** | **p value** |
| PEF AUC_0–24_ response | Placebo | 200 | -0.014 (0.059) |  |  |  |
|  | Olodaterol 2.5 BID | 99 | 0.627 (0.073) | 0.641 (0.063) | 0.516, 0.765 | < 0.0001 |
|  | Olodaterol 5 QD | 99 | 0.563 (0.073) | 0.577 (0.063) | 0.453, 0.701 | < 0.0001 |
|  | Olodaterol 5 BID | 100 | 0.653 (0.073) | 0.667 (0.063) | 0.544, 0.790 | < 0.0001 |
|  | Olodaterol 10 QD | 101 | 0.629 (0.073) | 0.643 (0.063) | 0.519, 0.766 | < 0.0001 |

Common study baseline mean (SE): 6.936 (0.146).
PEF: peak expiratory flow; AUC_0–24_: area under the curve from 0–24 hours; SE: standard error; CI: confidence interval; BID: twice daily; QD: once daily.

**Additional Table S4 Overall adjusted mean PEF and total Asthma Control Questionnaire score after 3 weeks.**

|  |  |  |  | **Difference from placebo** | | |
| --- | --- | --- | --- | --- | --- | --- |
| **End point** | **Treatment, µg** | **n** | **Mean response (SE)** | **Mean (SE)** | **95% CI** | **p value** |
| Mean morning PEF, L/min | Placebo | 201 | 395.36 (2.857) |  |  |  |
|  | Olodaterol 2.5 BID | 99 | 428.32 (3.586) | 32.957 (3.180) | 26.709, 39.206 | < 0.0001 |
|  | Olodaterol 5 QD | 100 | 427.99 (3.575) | 32.632 (3.167) | 26.409, 38.855 | < 0.0001 |
|  | Olodaterol 5 BID | 101 | 427.02 (3.562) | 31.660 (3.161) | 25.448, 37.872 | < 0.0001 |
|  | Olodaterol 10 QD | 101 | 427.26 (3.562) | 28.895 (3.161) | 22.683, 35.107 | < 0.0001 |
| Mean evening PEF, L/min | Placebo | 201 | 409.93 (2.717) |  |  |  |
|  | Olodaterol 2.5 BID | 99 | 438.80 (3.418) | 28.870 (3.046) | 22.884, 34.856 | < 0.0001 |
|  | Olodaterol 5 QD | 100 | 441.98 (3.407) | 32.056 (3.034) | 26.094, 38.018 | < 0.0001 |
|  | Olodaterol 5 BID | 101 | 441.74 (3.395) | 31.816 (3.028) | 25.864, 37.767 | < 0.0001 |
|  | Olodaterol 10 QD | 101 | 443.25 (3.395) | 33.327 (3.028) | 27.375, 39.278 | < 0.0001 |
| Total Asthma Control Questionnaire score | Placebo | 201 | 1.613 (0.040) |  |  |  |
|  | Olodaterol 2.5 BID | 99 | 1.256 (0.053) | -0.357 (0.053) | -0.461, -0.253 | < 0.0001 |
|  | Olodaterol 5 QD | 100 | 1.317 (0.053) | -0.296 (0.053) | -0.400, -0.192 | < 0.0001 |
|  | Olodaterol 5 BID | 101 | 1.312 (0.053) | -0.301 (0.053) | -0.405, -0.198 | < 0.0001 |
|  | Olodaterol 10 QD | 101 | 1.311 (0.053) | -0.302 (0.053) | -0.405, -0.198 | < 0.0001 |

Common study baseline mean (SE): 396.42 (8.867) for morning PEF, 413.02 (9.159) for evening PEF and 1.827 (0.053) for Asthma Control Questionnaire.
PEF: peak expiratory flow; SE: standard error; CI: confidence interval; BID: twice daily; QD: once daily.
